# Change in Serum Bilirubin Level as a Predictor of Incident Metabolic Syndrome

**DOI:** 10.1371/journal.pone.0168253

**Published:** 2016-12-09

**Authors:** You-Bin Lee, Seung-Eun Lee, Ji Eun Jun, Jae Hwan Jee, Ji Cheol Bae, Sang-Man Jin, Jae Hyeon Kim

**Affiliations:** 1 Division of Endocrinology and Metabolism, Department of Medicine, Samsung Medical Center, Sungkyunkwan University School of Medicine, Gangnam-gu, Seoul, Republic of Korea; 2 Department of Health Promotion Center, Samsung Medical Center, Sungkyunkwan University School of Medicine, Gangnam-gu, Seoul, Republic of Korea; 3 Division of Endocrinology and Metabolism, Department of Medicine, Samsung Changwon Hospital, Sungkyunkwan University School of Medicine, MasanHoiwon-gu, Changwon-si, Gyeongsangnam-do, Republic of Korea; 4 Department of Clinical Research Design and Evaluation, Samsung Advanced Institute for Health Sciences and Technology, Sungkyunkwan University, Gangnam-gu, Seoul, Republic of Korea; Chang Gung Memorial Hospital Kaohsiung Branch, TAIWAN

## Abstract

**Aim:**

Serum bilirubin level was negatively associated with the prevalence of metabolic syndrome (MetS) in previous cross-sectional studies. However, bilirubin variance preceding the development of MetS has yet to be investigated. We aimed to determine the effect of change in bilirubin concentration on the risk of incident MetS in healthy Korean adults.

**Methods:**

We conducted a retrospective longitudinal study of subjects who had undergone at least four yearly health check-ups between 2006 and 2012. Of 24,185 total individuals who received annual check-ups, 11,613 non-MetS participants with a baseline bilirubin level not exceeding 34.2 μmol/l were enrolled. We evaluated the association between percent change in bilirubin and risk of incident MetS.

**Results:**

During 55,407 person-years of follow-up, 2,439 cases of incident MetS developed (21.0%). Baseline serum bilirubin level clearly showed no association with the development of MetS in men but an independent significant inverse association in women which attenuated (hence may be mediated) by elevated homeostatic model assessment index 2 for insulin resistance (HOMA2-IR). However, increased risk for incident MetS was observed in higher percent change in bilirubin quartiles, with hazard ratios of 2.415 (95% CI 2.094–2.785) in men and 2.156 (95% CI 1.738–2.675) in women in the fourth quartile, compared to the lowest quartile, after adjusting for age, smoking status, medication history, alanine aminotransferase, uric acid, estimated glomerular filtration rate, fasting glucose, baseline diabetes mellitus prevalence, systolic blood pressure, waist circumference, and body mass index. The hazard ratios per one standard deviation increase in percent change in bilirubin as a continuous variable were 1.277 (95% CI 1.229–1.326) in men and 1.366 (95% CI 1.288–1.447) in women.

**Conclusions:**

Increases in serum bilirubin concentration were positively associated with a higher risk of incident MetS. Serum bilirubin increment might be a sensitive marker for the development of MetS.

## Introduction

Metabolic syndrome (MetS) is a constellation of interlinked metabolic conditions that seem to accelerate the development of cardiovascular disease [1–3]. Chronic inflammation, oxidative stress, and insulin resistance all play important roles in the initiation and advancement of MetS [1,3,4].

Bilirubin is not only the end product of heme catabolism, but also a potent endogenous anti-oxidant [5]. It also reflects serum antioxidant capacity [6]. Moreover, it exerts a protective effect against inflammatory processes in blood vessels [6,7]. In line with these findings, serum bilirubin concentration has been found to be inversely associated with oxidative stress-related disorders such as cardiovascular diseases, diabetes mellitus, obesity, and MetS [1,6,8–10]. In addition, a previous study reported an inverse association between mean bilirubin concentrations and the number of metabolic syndrome components in a population with atherogenic dyslipidemia [9].

However, most prior studies could not investigate the changes in bilirubin concentration preceding the development of MetS because of their cross-sectional nature. Heme oxygenase-1, an important enzyme in the production of bilirubin, which is highly inducible [6], increases in early phases of diabetes and decreases in late stages [11,12]. It is possible that bilirubin concentration increases in parallel with heme oxygenase-1 in the initiation phases of MetS and diabetes mellitus. However, studies have yet to explore change in bilirubin level as a marker of the development of MetS. Therefore, we investigated the effects of changes in bilirubin level on incident MetS development in healthy Korean adults.

## Materials and Methods

### 1. Study population and design

This was a retrospective longitudinal study. The study cohort comprised 24,185 participants who had undergone yearly health check-up programs for four or more years at the Health Promotion Center of Samsung Medical Center between January 2006 and December 2012. Among them, 7,285 subjects were excluded due to missing waist circumference measures. In addition, 4,816 people were excluded from analysis because they had MetS at baseline (n = 2,728), were diagnosed with MetS at the first follow-up visit (n = 1,516), had a baseline bilirubin level higher than 34.2 μmol/l (2.0 mg/dl) (n = 239), had hepatitis B (hepatitis B surface antigen presence) (n = 536) or hepatitis C (anti-hepatitis C virus antibody presence) (n = 111), had aspartate aminotransferase (AST) and/or alanine aminotransferase (ALT) levels higher than 80 U/l (n = 71), had missing baseline bilirubin level data (n = 2), or had an estimated glomerular filtration rate (eGFR) lower than 60 ml/min/1.73 m^2^ (n = 84). Therefore, 11,613 subjects (6,890 men and 4,723 women) with a mean age of 50.9 years (range: 18–89 years) were enrolled (S1 Fig). Subjects were observed until they were first diagnosed with MetS. If MetS did not develop until the final visit, the last visit was considered to be the end of follow-up. This study was approved by the Institutional Review Board (IRB) of Samsung Medical Center and was conducted in accordance with the Declaration of Helsinki.

### 2. Clinical and biochemical measurements

Medical history, smoking and alcohol consumption status, anthropometric data, and laboratory test results were collected during check-ups. Subjects were categorized by smoking status into never, past, and current smoker groups. They were also classified as drinkers and non-drinkers according to the response to the questionnaire asking whether they drink alcohol or not. Subjects were dichotomously categorized as drinkers and non-drinkers because information on the amount or frencies of alcohol consumption was not available. Participants were also required to check whether they take statin or not, and take aspirin or not by the questionnaires addressing these issues. Blood pressure (BP) was measured by trained nurses using mercury sphygmomanometers on patients’ right arms after at least five minutes of rest. Waist circumference (WC) was measured in the erect position between the costal margin and iliac crest at the end of expiration. Body mass index (BMI) was calculated as body weight in kilograms divided by height in meters squared (kg/m^2^). Estimated glomerular filtration rate (eGFR) was calculated according to the Modification of Diet in Renal Disease (MDRD) equation [13]. Insulin resistance, represented by the homeostatic model assessment index 2 for insulin resistance (HOMA2-IR), was derived utilizing the HOMA calculator (http://www.dtu.ox.ac.uk) [14].

Venous blood samples were obtained after an overnight fast and were sent to the central, certified laboratory in our center. Measurements included serum bilirubin, AST, ALT, fasting plasma glucose, plasma insulin, triglyceride (TG), low-density lipoprotein cholesterol (LDL-C), high density lipoprotein cholesterol (HDL-C), serum uric acid, and creatinine. The presence of hepatitis B surface antigen (HBs Ag) and anti-hepatitis C virus antibody (anti-HCV Ab) was also determined.

Serum bilirubin concentrations were measured using the diazonium salt/diazonium ion with blank method with Modular/Hitachi BIL-T reagents (Roche Diagnostics, Basel, Switzerland) on a Hitachi-7600, Modular DP-110 autoanalyzer (Hitachi, Tokyo, Japan). Plasma glucose levels were determined using the hexokinase method with Bayer Reagent Packs on an automated chemistry analyzer (Advia 1650 Autoanalyzer, Bayer Diagnostics, Leverkusen, Germany), and plasma insulin values were derived using an immunoradiometric assay (DIAsource Co., Louvain-la-Neuve, Belgium). Fasting HDL-C, LDL-C, and TG were measured using the enzymatic colorimetric method with a Modular D2400 (Roche Diagnostics, Basel, Switzerland).

### 3. Definitions

The definition of MetS was established according to the 2005 revision of the National Cholesterol Education Program Adult Treatment Panel III (NECP-ATP III) criteria, using Asian-specific cut-off values for abdominal obesity (WC ≥ 90 cm in men, ≥ 80 cm in women) [15].

Baseline bilirubin was defined as the serum bilirubin level measured on the first visit. The final bilirubin concentration was recorded one year before the date of diagnosis of MetS or at the final visit for subjects without a MetS diagnosis. Bilirubin change was derived by subtracting the final bilirubin level from the baseline bilirubin value. Percent change in bilirubin (PCB) was calculated according to the following formula: 100 (%) × [final bilirubin (μmol/l)–baseline bilirubin (μmol/l)]/[baseline bilirubin (μmol/l)]. Mean bilirubin level during the follow-up period was defined as the mean value of the bilirubin levels from the 2^nd^ to the final visit. Mean percent change in bilirubin (Mean PCB) was derived from the following equation: 100 (%) × [mean bilirubin level during the follow-up period (μmol/l)–baseline bilirubin (μmol/l)]/[baseline bilirubin (μmol/l)].

### 4. Statistical analyses

SPSS Software (Version 21, SPSS Inc., Chicago, IL, USA) was utilized for the statistical analyses. Characteristics of the study population according to development of MetS were analyzed separately for men and women. Continuous variables with normal distributions were compared using Student’s t-test and expressed as mean ± standard deviation, while continuous variables with uneven distributions were parallelized using Mann-Whitney U test and presented as median and interquartile range. For categorical variables, Pearson’s Chi-square test was performed, and frequencies and percentages were presented.

Multivariate Cox regression analysis was conducted to evaluate the hazard ratios (HRs) and 95% confidence intervals (CIs) for MetS incidence according to increases in baseline bilirubin level and PCB as continuous variables and quartiles. The HR for MetS incidence was also calculated according to one standard deviation increase in mean PCB as a continuous variable. These analyses were conducted separately for men and women, and sex-specific cutoff points were adopted for quartiles of baseline bilirubin and PCB in men and women. Cox regression models were adjusted for potential confounders. Model 1 was adjusted for age, smoking status, and medication history (whether taking statin or not, and taking aspirin or not). Model 2 was further adjusted for ALT, uric acid, eGFR, fasting glucose and prevalence of diabetes mellitus at the initial visit. Model 3 was additionally adjusted for systolic BP, WC and BMI. Moreover, alcohol history was further added as a potential confounder in Model 4 in those who answered the questionnaire regarding the current alcohol consumption status. Model 5 was adjusted for HOMA2-IR and all possible confounders previously mentioned except for the alcohol history and fasting glucose, in subjects whose fasting insulin level had been measured. Two-tailed probability values less than 0.05 were considered to be statistically significant.

Spearman’s rank correlation coefficients were used to explore the relationships between baseline bilirubin or PCB and anthropometric and biochemical parameters, which included variables possibly associated with MetS such as WC, BMI, BP, HOMA2-IR, TG, LDL-C, HDL-C, apolipoprotein A1 (apo A1), and apolipoprotein B (apo B).

## Results

### 1. Characteristics of the study population

During 55,407 person-years of follow-up, 1,695 cases in men and 744 cases in women of new MetS developed (21.0% in all subjects; 24.6% in men and 15.8% in women). The clinical characteristics and laboratory data of the study population according to the development of MetS are presented in Table 1. Subjects who developed MetS had higher WC, BMI, systolic and diastolic BP, fasting glucose, fasting insulin, HOMA2-IR, TG, LDL-C, AST, ALT, and serum uric acid values than those who did not. HDL-C level was significantly lower in those who were subsequently diagnosed with MetS. This was true for both male and female participants. Smoking status demonstrated a significant difference between the groups who did and did not develop MetS only in men (*P* < 0.001), while age varied significantly based on MetS development only in women (*P* < 0.001).

**Table 1:** Characteristics of Men and Women According to Development of Metabolic Syndrome.

	Men			Women		
	Incident metabolic syndrome [n (%)]			Incident metabolic syndrome [n (%)]		
	No	Yes	*P*- value	No	Yes	*P*-value
	5195 (75.4)	1695 (24.6)		3979 (84.2)	744 (15.8)	
Age (years)	52.0 ± 8.5	52.1 ± 8.1	0.521	48.8 ± 7.2	52.6 ± 7.7	< 0.001
Waist circumference (cm)	84.5 ± 6.0	89.1 ± 5.8	< 0.001	73.0 (69.0−77.0)	78.0 (75.0−83.0)	< 0.001
BMI (kg/m^2^)	23.55 ± 2.17	25.07 ± 2.09	< 0.001	21.73 ± 2.27	23.79 ± 2.54	< 0.001
Smoking status						
Current smoker [n (%)]	1302 (25.1)	535 (31.6)	<0.001	71 (1.8)	9 (1.2)	0.088
Never-smoker [n (%)]	1484 (28.6)	379 (22.4)		3776 (94.9)	720 (96.8)	
Systolic BP (mmHg)	110.0 (101.0−120.0)	114.0 (105.0−124.0)	< 0.001	106.0 (98.0−116.0)	114.0 (104.0−125.0)	< 0.001
Diastolic BP (mmHg)	70.0 (62.0−76.0)	71.0 (65.0−78.0)	< 0.001	64.0 (60.0−71.0)	70.0 (61.0−75.0)	< 0.001
eGFR (ml/min/1.73 m^2^)	87.82 ± 11.50	87.39 ± 11.83	0.192	90.75 ± 12.47	89.14 ± 12.89	0.001
Fasting glucose (mmol/l)	4.83 (4.55−5.16)	5.00 (4.66−5.38)	< 0.001	4.66 (4.44−5.00)	4.88 (4.61−5.22)	< 0.001
Fasting insulin (pmol/l)	52.78 (40.98−67.37)^a^	61.81 (48.62−79.17)^b^	< 0.001	53.48 (41.67−65.98)^c^	61.81 (49.31−78.48)^d^	< 0.001
HOMA2-IR	1.0 (0.8−1.3)^a^	1.2 (0.9−1.5)^b^	< 0.001	1.0 (0.8−1.2)^c^	1.1 (0.9−1.4)^d^	< 0.001
Total cholesterol (mmol/l)	4.88 ± 0.79	4.92 ± 0.79	0.052	4.94 ± 0.84	5.13 ± 0.92	< 0.001
TG (mmol/l)	1.14 (0.87−1.51)	1.49 (1.16−2.00)	< 0.001	0.90 (0.70−1.18)	1.22 (0.95−1.57)	< 0.001
LDL-C (mmol/l)	3.19 ± 0.71	3.29 ± 0.72	< 0.001	3.06 ± 0.73	3.41 ± 0.82	< 0.001
HDL-C (mmol/l)	1.48 ± 0.32	1.33 ± 0.27	< 0.001	1.71 ± 0.35	1.51 ± 0.31	< 0.001
AST (U/l)	22.0 (19.0−26.0)	23.0 (19.0−27.0)	< 0.001	19.0 (17.0−23.0)	20.0 (17.0−24.0)	< 0.001
ALT (U/l)	20.0 (16.0−27.0)	23.0 (18.0−31.0)	< 0.001	15.0 (12.0−19.0)	17.0 (14.0−22.0)	< 0.001
Serum uric acid (μmol/l)	340.85 ± 66.03	358.10 ± 74.36	< 0.001	239.13 ± 48.78	262.92 ± 52.94	< 0.001
Baseline bilirubin (μmol/l)	16.8 ± 5.7	16.6 ± 5.6	0.332	13.5 ± 5.0	12.7 ± 5.1	< 0.001
Final bilirubin (μmol/l)	15.2 ± 5.6	17.4 ± 6.5	< 0.001	11.4 ± 4.4	12.7 ± 4.9	< 0.001
Percent change in bilirubin (%)	-11.1 (-26.7−11.1)	0.0 (-16.7−28.6)	< 0.001	-15.4 (-33.3−0.0)	0.0 (-20.0−25.0)	< 0.001

Continuous variables with normal distributions are expressed as mean ± standard deviation, whereas continuous variables with non-normal distributions are expressed as median (interquartile range).

Abbreviations: BMI, body mass index; BP, blood pressure; eGFR, estimated glomerular filtration rate; HOMA2-IR, homeostasis model assessment index 2 for insulin resistance; TG, triglyceride; LDL-C, low-density lipoprotein cholesterol; HDL-C, high-density lipoprotein cholesterol; AST, aspartate aminotransferase; ALT, alanine aminotransferase.

^a^ n = 3502,

^b^ n = 1036,

^c^ n = 2012,

^d^ n = 358

In men, there was no significant difference in baseline bilirubin concentration between the two groups. In women, baseline bilirubin level in those who developed MetS was lower than that in those who did not (*P* < 0.001). However, PCB was significantly lower (more negative) in participants who remained free of MetS than in those who developed MetS (*P* < 0.001) in individuals of both sexes.

### 2. Baseline bilirubin level and incident metabolic syndrome

The HRs and 95% CIs for incident MetS with respect to quartile of baseline bilirubin concentration and by one standard deviation increase in baseline bilirubin level as a continuous variable in men and women were calculated (Tables 2 and 3). Baseline bilirubin level as a continuous variable and its quartiles demonstrated no significant effect on the risk of incident MetS regardless of adjustment for potential confounders in men. In women, incidence of MetS tended to decrease as the baseline bilirubin quartile advanced. This association was significant in Model 1–4 when multivariate Cox regression analysis was performed with adjustment for potential confounders (Table 3). In women, the HRs for MetS incidence by the increase in baseline bilirubin concentration as a continuous variable also showed an inverse trend in Model 1–4 albeit it was not significant in Model 1 and 3. This inverse association was markedly attenuated when HOMA2-IR was added as a potential confounder in Model 5.

**Table 2:** Hazard Ratios and 95% Confidence Intervals for Incident Metabolic Syndrome according to Baseline Bilirubin Level as a Continuous Variable, and Baseline Bilirubin Quartile (Q1-Q4) in Men (n = 6890).

	Quartiles of baseline bilirubin					Continuous variable HR (95% CI) per 1 standard deviation^a^	*P*-value
	Q1 (n = 1789)	Q2 (n = 1841)	Q3 (n = 1924)	Q4 (n = 1336)	*P* for trend		
	3.4−12.0 μmol/l	13.7−15.4 μmol/l	17.1−20.5 μmol/l	22.2−34.2 μmol/l			
	(0.2−0.7 mg/dl)	(0.8−0.9 mg/dl)	(1.0−1.2 mg/dl)	(1.3−2.0 mg/dl)			
Mean baseline bilirubin (μmol/l)	10.4±1.7	14.5±0.9	18.6±1.4	25.7±3.4			
Incidence of MetS	442/1789 (24.7%)	465/1841 (25.3%)	471/1924 (24.5%)	317/1336 (23.7%)	0.472		
Model 1	1(ref.)	1.069 (0.938–1.218)	1.033 (0.907–1.177)	1.043 (0.902–1.207)	0.663	1.004 (0.957–1.054)	0.859
Model 2	1(ref.)	1.035 (0.909–1.180)	1.006 (0.883–1.146)	1.017 (0.879–1.176)	0.926	1.000 (0.952–1.049)	0.991
Model 3	1(ref.)	1.020 (0.895–1.162)	1.005 (0.881–1.145)	0.966 (0.835–1.119)	0.661	0.992 (0.944–1.041)	0.734
Model 4	1(ref.)	1.053 (0.910–1.220)	1.071 (0.924–1.240)	0.982 (0.833–1.159)	0.999	1.003 (0.950–1.060)	0.906
Model 5	1(ref.)	1.077 (0.907–1.279)	1.063 (0.896–1.260)	0.992 (0.821–1.199)	0.961	0.991 (0.932–1.055)	0.786

Model 1: adjusted for age, smoking status, and medication (statin and aspirin)

Model 2: adjusted for age, smoking status, medication (statin and aspirin), ALT, uric acid, eGFR, fasting glucose, and baseline diabetes mellitus prevalence

Model 3: adjusted for age, smoking status, medication (statin and aspirin), ALT, uric acid, eGFR, fasting glucose, baseline diabetes mellitus prevalence, systolic BP, waist circumference, and BMI

Model 4: adjusted for age, smoking status, medication (statin and aspirin), ALT, uric acid, eGFR, fasting glucose, baseline diabetes mellitus prevalence, systolic BP, waist circumference, BMI, and alcohol history (n = 5542)

Model 5: adjusted for age, smoking status, medication (statin and aspirin), ALT, uric acid, eGFR, baseline diabetes mellitus prevalence, systolic BP, waist circumference, BMI, and HOMA2-IR (n = 4538)

Abbreviations: HR, hazard ratio; CI, confidence interval; MetS, metabolic syndrome; ALT, alanine aminotransferase; eGFR, estimated glomerular filtration rate; BP, blood pressure; BMI, body mass index; HOMA2-IR, homeostasis model assessment index 2 for insulin resistance.

^a^ 1 standard deviation = 5.64 μmol/l

**Table 3:** Hazard Ratios and 95% Confidence Intervals for Incident Metabolic Syndrome according to Baseline Bilirubin Level as a Continuous Variable, and Baseline Bilirubin Quartile (Q1-Q4) in Women (n = 4723).

	Quartiles of baseline bilirubin					Continuous variable HR (95% CI) per 1 standard deviation^a^	*P*-value
	Q1 (n = 980)	Q2 (n = 1532)	Q3 (n = 1127)	Q4 (n = 1084)	*P* for trend		
	3.4−8.6 μmol/l	10.3−12.0 μmol/l	13.7−15.4 μmol/l	17.1−34.2 μmol/l			
	(0.2−0.5 mg/dl)	(0.6−0.7 mg/dl)	(0.8−0.9 mg/dl)	(1.0−2.0 mg/dl)			
Mean baseline bilirubin (μmol/l)	7.7±1.2	11.1±0.9	14.4±0.8	20.6±4.0			
Incidence of MetS	208/980 (21.2%)	235/755 (15.3%)	153/1127 (13.6%)	148/1084 (13.7%)	< 0.001		
Model 1	1(ref.)	0.744 (0.617–0.897)	0.740 (0.600–0.913)	0.776 (0.628–0.960)	0.023	0.936 (0.867–1.011)	0.092
Model 2	1(ref.)	0.722 (0.599–0.871)	0.714 (0.579–0.882)	0.715 (0.577–0.886)	0.003	0.908 (0.840–0.982)	0.016
Model 3	1(ref.)	0.769 (0.637–0.928)	0.730 (0.591–0.902)	0.767 (0.619–0.951)	0.013	0.929 (0.859–1.004)	0.063
Model 4	1(ref.)	0.782 (0.633–0.965)	0.778 (0.617–0.981)	0.746 (0.584–0.952)	0.022	0.908 (0.831–0.992)	0.032
Model 5	1(ref.)	0.761 (0.570–1.016)	0.983 (0.725–1.334)	0.915 (0.671–1.248)	0.911	1.023 (0.922–1.136)	0.667

Model 1: adjusted for age, smoking status, and medication (statin and aspirin)

Model 2: adjusted for age, smoking status, medication (statin and aspirin), ALT, uric acid, eGFR, fasting glucose, and baseline diabetes mellitus prevalence

Model 3: adjusted for age, smoking status, medication (statin and aspirin), ALT, uric acid, eGFR, fasting glucose, baseline diabetes mellitus prevalence, systolic BP, waist circumference, and BMI

Model 4: adjusted for age, smoking status, medication (statin and aspirin), ALT, uric acid, eGFR, fasting glucose, baseline diabetes mellitus prevalence, systolic BP, waist circumference, BMI, and alcohol history (n = 3936)

Model 5: adjusted for age, smoking status, medication (statin and aspirin), ALT, uric acid, eGFR, baseline diabetes mellitus prevalence, systolic BP, waist circumference, BMI, and HOMA2-IR (n = 2368)

Abbreviations: HR, hazard ratio; CI, confidence interval; MetS, metabolic syndrome; ALT, alanine aminotransferase; eGFR, estimated glomerular filtration rate; BP, blood pressure; BMI, body mass index; HOMA2-IR, homeostasis model assessment index 2 for insulin resistance.

^a^ 1 standard deviation = 5.02 μmol/l

### 3. Percent change in bilirubin (PCB) and incident metabolic syndrome

HRs and 95% CIs for incident MetS according to PCB quartile and one standard deviation increment in PCB as a continuous variable were presented separately for men (Table 4) and women (Table 5). In all models (Model 1–5) adjusted for possible confounding factors, risk for incident MetS increased significantly in higher quartile groups. In men, the HR per one standard deviation increase in PCB as a continuous variable was 1.277 (95% CI, 1.229–1.326; *P* < 0.001) in Model 3 (Table 4), and in women, it was estimated to be 1.366 (95% CI, 1.288–1.447; *P* < 0.001) in Model 3 (Table 5). Increase in mean PCB as a continuous variable was also significantly associated with increased risk for incident MetS in both sex group (S1 Table).

**Table 4:** Hazard Ratios and 95% Confidence Intervals for Incident Metabolic Syndrome according to Percent Change in Bilirubin Level as a Continuous Variable, and Percent Change in Bilirubin Quartile (Q1-Q4) in Men (n = 6890).

	Quartiles of percent change in bilirubin					Continuous variable HR (95% CI) per 1 standard deviation^a^	*P*-value
	Q1 (n = 1796)	Q2 (n = 1566)	Q3 (n = 1828)	Q4 (n = 1700)	*P* for trend		
	-75−-25%	-24−-10%	-9−14%	15−275%			
Mean PCB (%)	-36.40±9.78	-16.18±4.26	2.88±6.87	46.69±33.73			
Incidence of MetS	275/1796 (15.3%)	321/1566 (20.5%)	474/1828 (25.9%)	625/1700 (36.8%)	< 0.001		
Model 1	1(ref.)	1.341 (1.141–1.575)	1.768 (1.524–2.052)	2.678 (2.323–3.086)	< 0.001	1.321 (1.273–1.370)	< 0.001
Model 2	1(ref.)	1.333 (1.134–1.566)	1.759 (1.515–2.042)	2.710 (2.351–3.123)	< 0.001	1.328 (1.280–1.378)	< 0.001
Model 3	1(ref.)	1.259 (1.071–1.479)	1.655 (1.426–1.922)	2.415 (2.094–2.785)	< 0.001	1.277 (1.229–1.326)	< 0.001
Model 4	1(ref.)	1.269 (1.062–1.517)	1.676 (1.419–1.979)	2.359 (2.013–2.765)	< 0.001	1.278 (1.225–1.333)	< 0.001
Model 5	1(ref.)	1.254 (1.026–1.533)	1.516 (1.255–1.831)	2.339 (1.954–2.801)	< 0.001	1.287 (1.224–1.353)	< 0.001

Model 1: adjusted for age, smoking status, and medication (statin and aspirin)

Model 2: adjusted for age, smoking status, medication (statin and aspirin), ALT, uric acid, eGFR, fasting glucose, and baseline diabetes mellitus prevalence

Model 3: adjusted for age, smoking status, medication (statin and aspirin), ALT, uric acid, eGFR, fasting glucose, baseline diabetes mellitus prevalence, systolic BP, waist circumference, and BMI

Model 4: adjusted for age, smoking status, medication (statin and aspirin), ALT, uric acid, eGFR, fasting glucose, baseline diabetes mellitus prevalence, systolic BP, waist circumference, BMI, and alcohol history (n = 5542)

Model 5: adjusted for age, smoking status, medication (statin and aspirin), ALT, uric acid, eGFR, baseline diabetes mellitus prevalence, systolic BP, waist circumference, BMI, and HOMA2-IR (n = 4538)

Abbreviations: HR, hazard ratio; CI, confidence interval; PCB, percent change in bilirubin; MetS, metabolic syndrome; ALT, alanine aminotransferase; eGFR, estimated glomerular filtration rate; BP, blood pressure; BMI, body mass index; HOMA2-IR, homeostasis model assessment index 2 for insulin resistance.

^a^ 1 standard deviation = 35.59%

**Table 5:** Hazard Ratios and 95% Confidence Intervals for Incident Metabolic Syndrome according to Percent Change in Bilirubin Level as a Continuous Variable, and Percent Change in Bilirubin Quartile (Q1-Q4) in Women (n = 4723).

	Quartiles of percent change in bilirubin					Continuous variable HR (95% CI) per 1 standard deviation^a^	*P*-value
	Q1 (n = 1179)	Q2 (n = 1039)	Q3 (n = 1338)	Q4 (n = 1167)	*P* for trend		
	-80−-31%	-30−-15%	-14−11%	12−267%			
Mean PCB (%)	-44.01±9.92	-22.77±4.57	-3.97±6.94	39.62±30.54			
Incidence of MetS	117/1179 (9.9%)	107/1039 (10.3%)	208/1338 (15.5%)	312/1167 (26.7%)	< 0.001		
Model 1	1(ref.)	0.942 (0.724–1.224)	1.333 (1.062–1.674)	2.390 (1.929–2.962)	< 0.001	1.353 (1.283–1.428)	< 0.001
Model 2	1(ref.)	0.879 (0.674–1.146)	1.343 (1.069–1.687)	2.445 (1.973–3.031)	< 0.001	1.384 (1.311–1.462)	< 0.001
Model 3	1(ref.)	0.906 (0.695–1.182)	1.317 (1.048–1.656)	2.156 (1.738–2.675)	< 0.001	1.366 (1.288–1.447)	< 0.001
Model 4	1(ref.)	0.888 (0.662–1.190)	1.286 (0.999–1.655)	2.093 (1.650–2.656)	< 0.001	1.358 (1.273–1.448)	< 0.001
Model 5	1(ref.)	1.120 (0.773–1.624)	1.489 (1.071–2.070)	2.088 (1.518–2.872)	< 0.001	1.299 (1.196–1.410)	< 0.001

Model 1: adjusted for age, smoking status, and medication (statin and aspirin)

Model 2: adjusted for age, smoking status, medication (statin and aspirin), ALT, uric acid, eGFR, fasting glucose, and baseline diabetes mellitus prevalence

Model 3: adjusted for age, smoking status, medication (statin and aspirin), ALT, uric acid, eGFR, fasting glucose, baseline diabetes mellitus prevalence, systolic BP, waist circumference, and BMI

Model 4: adjusted for age, smoking status, medication (statin and aspirin), ALT, uric acid, eGFR, fasting glucose, baseline diabetes mellitus prevalence, systolic BP, waist circumference, BMI, and alcohol history (n = 3936)

Model 5: adjusted for age, smoking status, medication (statin and aspirin), ALT, uric acid, eGFR, baseline diabetes mellitus prevalence, systolic BP, waist circumference, BMI, and HOMA2-IR (n = 2368)

Abbreviations: HR, hazard ratio; CI, confidence interval; MetS, metabolic syndrome; ALT, alanine aminotransferase; eGFR, estimated glomerular filtration rate; BP, blood pressure; BMI, body mass index; HOMA2-IR, homeostasis model assessment index 2 for insulin resistance.

^a^ 1 standard devidation = 34.78%

**Fig 1:**
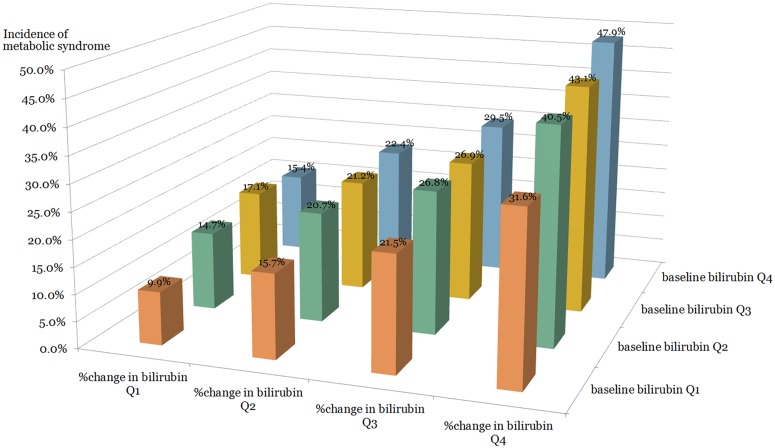
The incidence of metabolic syndrome according to baseline bilirubin and percent change in bilirubin quartiles in men.

**Fig 2:**
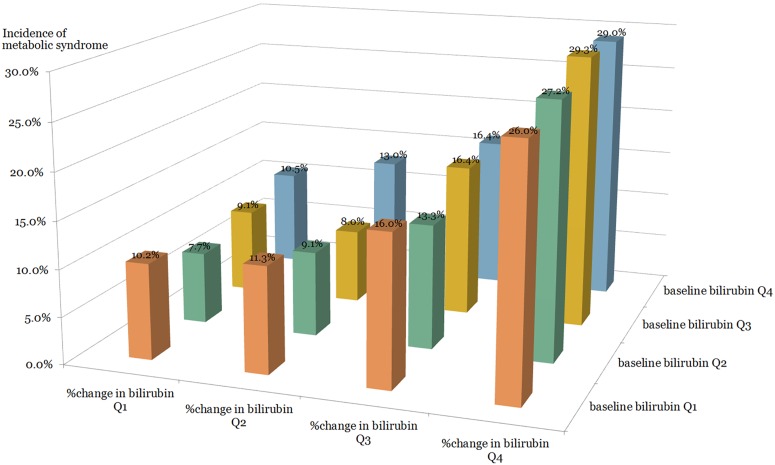
The incidence of metabolic syndrome according to baseline bilirubin and percent change in bilirubin quartiles in women.

The incidence of MetS was calculated separately in 16 subgroups divided into quartiles of baseline bilirubin and PCB (Figs 1 and 2). In the same quartile group of baseline bilirubin, the incidence of MetS increased sequentially as the PCB quartile advanced, in both sexes (Figs 1 and 2).

### 4. Correlations between baseline bilirubin, percent change in bilirubin, and other parameters

Correlations between baseline bilirubin or PCB and anthropometric and biochemical parameters are presented in Table 6. Baseline bilirubin level was negatively correlated with bilirubin change (rho = -0.462, *P* < 0.001) and PCB (rho = -0.389, *P* < 0.001). Baseline bilirubin concentration was negatively correlated with age, fasting insulin, and HOMA2-IR, while PCB was positively correlated with the same variables. Baseline bilirubin level was positively correlated with LDL-C, apo A1, and apo B, although PCB was negatively correlated with these parameters.

**Table 6:** Correlations between Baseline Bilirubin or Percent Change in Bilirubin and Anthropometric and Biochemical Parameters.

	Baseline bilirubin (mg/dl)			Percent change in bilirubin (%)	
Variables	Rank correlation coefficient (rho)	*P*	Variables	Rank correlation coefficient (rho)	*P*
Age (years)	-0.023	0.013	Age (years)	0.083	< 0.001
Waist circumference (cm)	0.181	< 0.001	Waist circumference (cm)	0.146	< 0.001
BMI (kg/m^2^)	0.089	< 0.001	BMI (kg/m^2^)	0.147	< 0.001
Systolic BP (mmHg)	0.099	< 0.001	Systolic BP (mmHg)	0.029	0.002
Diastolic BP (mmHg)	0.131	< 0.001	Diastolic BP (mmHg)	0.021	0.022
eGFR (ml/min/1.73 m^2^)	-0.054	< 0.001	eGFR (ml/min/1.73 m^2^)	-0.023	0.015
Fasting glucose (mmol/l)	0.079	< 0.001	Fasting glucose (mmol/l)	0.013	0.155
Fasting insulin (pmol/l) ^a^	-0.087	< 0.001	Fasting insulin (pmol/l) ^a^	0.115	< 0.001
HOMA2-IR ^a^	-0.081	< 0.001	HOMA2-IR ^a^	0.113	< 0.001
TG (mmol/l)	0.018	0.057	TG (mmol/l)	0.114	< 0.001
LDL-C (mmol/l)	0.028	0.003	LDL-C (mmol/l)	-0.024	0.009
HDL-C (mmol/l)	-0.035	< 0.001	HDL-C (mmol/l)	-0.136	< 0.001
Apo A1	0.140	< 0.001	Apo A1	-0.082	< 0.001
Apo B	0.142	< 0.001	Apo B	-0.062	< 0.001
AST (U/l)	0.094	< 0.001	AST (U/l)	0.027	0.004
ALT (U/l)	0.127	< 0.001	ALT (U/l)	0.070	< 0.001
Serum uric acid (μmol/l)	0.214	< 0.001	Serum uric acid (μmol/l)	0.082	< 0.001
Bilirubin change (μmol/l)	-0.462	< 0.001	Baseline bilirubin (μmol/l)	-0.389	< 0.001

Abbreviations: BMI, body mass index; BP, blood pressure; eGFR, estimated glomerular filtration rate; HOMA2-IR, homeostasis model assessment index 2 for insulin resistance; HOMA2-%B, homeostasis model assessment index 2 for pancreatic beta-cell function; TG, triglyceride; LDL-C, low-density lipoprotein cholesterol; HDL-C, high-density lipoprotein cholesterol; Apo A1, Apolipoprotein A1; Apo B, Apolipoprotein B; AST, aspartate aminotransferase; ALT, alanine aminotransferase

^a^ n = 6908

## Discussion

To the best of our knowledge, this was the first study investigating whether change in serum bilirubin concentration, rather than baseline serum bilirubin level, is associated with the risk of incident MetS in a longitudinal design. This longitudinal study demonstrated a significant association between change in bilirubin level and the development of MetS in both genders, even in fully-adjusted models. Association between baseline bilirubin level and the risk of incident MetS, which has been reported by previous studies, was confirmed only in some multivariate analysis conducted in female subjects.

Previous studies have consistently reported that serum bilirubin level is inversely correlated with cardiovascular diseases, hypertension, diabetes mellitus, obesity, and MetS [6,10]. Certain genotype allele carriers with higher bilirubin concentrations showed an inverse association with cardiovascular diseases risk in the Framingham Heart Study population [8], and a lower coronary heart disease risk was reported in women with higher bilirubin level in a prospective study with 4 year’s follow-up [16]. In our data, baseline bilirubin level showed a significant inverse association with MetS incidence only in women.

In relation to these findings, the possible antioxidant activity of bilirubin on low-density lipoprotein and consequent inhibition of the atherosclerotic process have been suggested in prior reports [17]. However, most previous reports did not explore changes in bilirubin concentration in the early phases of MetS, nor preceding the development of this disease [1,2]. The majority of recent studies showing a negative association between bilirubin and MetS were cross-sectional. Although Lee et al. demonstrated a significant negative relationship between baseline bilirubin concentration at the initial visit and incident MetS in a two-point longitudinal design [1], changes in bilirubin levels were not taken into account. Male subjects in that study were examined in 2007 and re-evaluated only in 2011, which resulted in a gap of four years between the initial visit and follow-up. Therefore, a discrepancy between the development of MetS and the detection of it could exist, and data regarding changes in serum bilirubin concentration preceding the development of MetS are not yet available.

Although the retrospective design of this study limits the exploration of cause-and-effect relationships and mechanisms, positive values of PCB may indicate a possible increase in oxidative stress preceding the occurrence of MetS.

Bilirubin is produced through the action of heme oxygenase [6], the rate-limiting enzyme in the catabolism of heme [5]. Formation of bilirubin is inhibited by the down-regulation of heme oxygenase activity [18]. Heme oxygenase exists as two isoforms, heme oxygenase-1 and heme oxygenase-2 [6]. Of them, heme oxygenase-1 is highly inducible in response to a variety of chemical and physical stressors including hypoxia, hyperoxia, hyperglycemia and pro-inflammatory cytokines [6], and it is considered to be a reliable and sensitive marker of cell stress [11,19]. Heme oxygenase-1 also contributes to metabolic control in both *in vitro* and *in vivo* models [20]. High glucose exposure leads to induction of heme oxygenase-1 gene expression and enzyme activity in islets in accordance with elevation in intracellular peroxide concentration [6,21,22], while chronic hyperglycemia results in a decrease in heme oxygenase activity in rat models [23,24]. Also, dissimilar expression of heme oxygenase-1 according to diabetes stage has been found, which indicates an increase in heme oxygenase-1 level in the early phases and a subsequent decrease in the late stages [11,12]. Bao et al demonstrated that increased plasma heme oxygenase-1 level was associated with higher odds ratios for new type 2 diabetes mellitus in a case control study [11], suggesting that the plasma level of heme oxygenase-1 is a reflection of oxidative stress generated prior to the development of diabetes mellitus [6]. The aforementioned studies proposed that heme oxygenase is induced in the early phases and decreased in the late stages of diseases related to glucose metabolism. It is highly likely that the bilirubin concentration changes according to disease course in parallel with heme oxygenase-1 expression. A recent study showing that the duration of type 2 diabetes is inversely associated with serum bilirubin levels within physiologic range [25] also corresponds to this suggestion. Additional prospective studies exploring serial changes of bilirubin and heme oxygenase-1 concentrations preceding and following the development of MetS may further support this speculation.

Positive change in bilirubin might be a sensitive biomarker for the development of MetS in the early phases of the process. Also, an increase in bilirubin may be a surrogate marker representing the body’s response to stressors such as reactive oxygen species in these initial stages. In our data, baseline bilirubin, which showed a trend of inverse association with MetS incidence in women of our data and has been consistently proposed as a protective factor of MetS [1,6], was negatively correlated with bilirubin change and PCB while PCB showed a statistically significant association with incident MetS.

Age, fasting insulin, and HOMA2-IR were negatively correlated with baseline bilirubin level, and they were positively correlated with PCB. On the other hand, LDL-C, apo A1, and apo B levels were positively correlated with baseline bilirubin concentration while they were inversely correlated with PCB. We speculate that these findings may support the idea that changes in lipoprotein[Lp](a) might be related to the underlying mechanism. Lp(a) mediates systemic inflammatory process and endothelial dysfunction, and acts as one of the cardiovascular risk factors, and it utilizes atherogenic and prothrombotic pathways [26,27]. Bilirubin exerts antioxidant activity on low-density lipoprotein [17,28,29]. Oxidation of low-density lipoprotein lipids may result in oxidative modification of the lipoprotein’s protein moiety, apoprotein B-100, which is a structural protein for LDL, and Lp(a) [17,28,29]. Oxidation of phospholipids including Lp(a) particles causes damages in epitopes which inhibit them from being captured by the immunoassay, and leading to an apparently low Lp(a) concentration [30]. If oxidation of Lp(a) is also reduced by bilirubin, the apparent Lp(a) concentration will not be decreased subsequently. Onat A et al. reported that Lp(a) was positively associated with total cholesterol, and negatively associated with fasting insulin [26]. The directions of the correlation of Lp(a) with these variables seem to be in concordance with those of baseline bilirubin.

Our study has several strengths. As a large longitudinal study, a substantial number of subjects of both sexes were enrolled, and a considerable number of cases of incident MetS were observed. In addition, we verified the development of MetS through annual follow-up. To the best of our knowledge, this is the first investigation of change in bilirubin level as a factor affecting initiation of MetS.

However, there are some limitations to the present study. First, we analyzed data from participants who voluntarily visited a health promotion center and underwent annual follow-up four or more times; this group might not be representative of the general population. In addition, we never measured plasma heme oxygenase-1 concentration, which might have provided direct evidence regarding the relationship between bilirubin and MetS. Also, clarification of the causal relationships and mechanisms may be limited by the retrospective design of this study. Finally, we evaluated the association between PCB and new-onset MetS after adjusting for potential confounders such as smoking status, WC, BMI, and systolic BP, and only information collected at the initial visit was utilized for confounders. Thus, changes in medical condition and the influence of life-style modification were not taken into account.

## Conclusions

In conclusion, an increase in bilirubin concentration, defined as PCB, was positively associated with incident MetS in a healthy Korean population, indicating that bilirubin increase might precede the development of MetS. Positive levels of PCB may reflect an increase in oxidative stress preceding new-onset MetS, and further studies are needed to confirm these suggestions.

## Supporting Information

S1 Fig Enrollment, exclusions, and follow-up. Abbreviations: HBs Ag, hepatitis B surface antigen; anti-HCV Ab, anti-hepatitis C virus antibody; AST, aspartate aminotransferase; ALT, alanine aminotransferase; eGFR, estimated glomerular filtration rate.
(TIF)

S1 Table Hazard Ratios and 95% Confidence Intervals for Incident Metabolic Syndrome according to Mean Percent Change in Bilirubin Level as a Continuous Variable in Men and Women. (DOCX)

## Abbreviations

ALT: alanine aminotransferase
anti-HCV Ab: anti-hepatitis C virus antibody
apo A1: apolipoprotein A1
apo B: apolipoprotein B
AST: aspartate aminotransferase
BMI: body mass index
BP: blood pressure
CI: confidence interval
eGFR: estimated glomerular filtration rate
HBs Ag: hepatitis B surface antigen
HDL-C: high density lipoprotein cholesterol
HOMA2-IR: homeostatic model assessment index 2 for insulin resistance
HR: hazard ratio
LDL-C: low-density lipoprotein cholesterol
MetS: metabolic syndrome
PCB: percent change in bilirubin
TG: triglyceride
WC: waist circumference

## References

[1] Lee MJ, Jung CH, Kang YM, Hwang JY, Jang JE, Leem J, et al Serum bilirubin as a predictor of incident metabolic syndrome: a 4-year retrospective longitudinal study of 6205 initially healthy Korean men. Diabetes Metab. 2014;40: 305–309. 10.1016/j.diabet.2014.04.006 24951082

[2] Oda E, Aizawa Y. Total bilirubin is inversely associated with metabolic syndrome but not a risk factor for metabolic syndrome in Japanese men and women. Acta Diabetol. 2013;50: 417–422. 10.1007/s00592-012-0447-5 23224110

[3] Rutter MK, Meigs JB, Sullivan LM, D'Agostino RB Sr., Wilson PW. C-reactive protein, the metabolic syndrome, and prediction of cardiovascular events in the Framingham Offspring Study. Circulation. 2004;110: 380–385. 10.1161/01.CIR.0000136581.59584.0E 15262834

[4] Ota T. Chemokine systems link obesity to insulin resistance. Diabetes Metab J. 2013;37: 165–172. 10.4093/dmj.2013.37.3.165 23807918 PMC3689012

[5] Kapitulnik J. Bilirubin: an endogenous product of heme degradation with both cytotoxic and cytoprotective properties. Mol Pharmacol. 2004;66: 773–779. 10.1124/mol.104.002832 15269289

[6] Vitek L. The role of bilirubin in diabetes, metabolic syndrome, and cardiovascular diseases. Front Pharmacol. 2012;3: 55 10.3389/fphar.2012.00055 22493581 PMC3318228

[7] Kawamura K, Ishikawa K, Wada Y, Kimura S, Matsumoto H, Kohro T, et al Bilirubin from heme oxygenase-1 attenuates vascular endothelial activation and dysfunction. Arterioscler Thromb Vasc Biol. 2005;25: 155–160. 10.1161/01.ATV.0000148405.18071.6a 15499042

[8] Lin JP, O'Donnell CJ, Schwaiger JP, Cupples LA, Lingenhel A, Hunt SC, et al Association between the UGT1A1*28 allele, bilirubin levels, and coronary heart disease in the Framingham Heart Study. Circulation. 2006;114: 1476–1481. 10.1161/CIRCULATIONAHA.106.633206 17000907

[9] Giral P, Ratziu V, Couvert P, Carrie A, Kontush A, Girerd X, et al Plasma bilirubin and gamma-glutamyltransferase activity are inversely related in dyslipidemic patients with metabolic syndrome: relevance to oxidative stress. Atherosclerosis. 2010;210: 607–613. 10.1016/j.atherosclerosis.2009.12.026 20129611

[10] Kunutsor SK, Bakker SJ, Gansevoort RT, Chowdhury R, Dullaart RP. Circulating total bilirubin and risk of incident cardiovascular disease in the general population. Arterioscler Thromb Vasc Biol. 2015;35: 716–724. 10.1161/ATVBAHA.114.304929 25593130

[11] Bao W, Song F, Li X, Rong S, Yang W, Zhang M, et al Plasma heme oxygenase-1 concentration is elevated in individuals with type 2 diabetes mellitus. PLoS One. 2010;5: e12371 10.1371/journal.pone.0012371 20811623 PMC2928270

[12] Song F, Qi X, Chen W, Jia W, Yao P, Nussler AK, et al Effect of Momordica grosvenori on oxidative stress pathways in renal mitochondria of normal and alloxan-induced diabetic mice. Involvement of heme oxygenase-1. Eur J Nutr. 2007;46: 61–69. 10.1007/s00394-006-0632-9 17278042

[13] Levey AS, Bosch JP, Lewis JB, Greene T, Rogers N, Roth D. A more accurate method to estimate glomerular filtration rate from serum creatinine: a new prediction equation. Modification of Diet in Renal Disease Study Group. Ann Intern Med. 1999;130: 461–470 10075613 10.7326/0003-4819-130-6-199903160-00002

[14] Levy JC, Matthews DR, Hermans MP. Correct homeostasis model assessment (HOMA) evaluation uses the computer program. Diabetes Care. 1998;21: 2191–2192 9839117 10.2337/diacare.21.12.2191

[15] Grundy SM, Cleeman JI, Daniels SR, Donato KA, Eckel RH, Franklin BA, et al Diagnosis and management of the metabolic syndrome: an American Heart Association/National Heart, Lung, and Blood Institute Scientific Statement. Circulation. 2005;112: 2735–2752. 10.1161/CIRCULATIONAHA.105.169404 16157765

[16] Onat A, Can G, Ornek E, Cicek G, Ayhan E, Dogan Y. Serum gamma-glutamyltransferase: independent predictor of risk of diabetes, hypertension, metabolic syndrome, and coronary disease. Obesity (Silver Spring). 2012;20: 842–848. 21633402 10.1038/oby.2011.136

[17] Breimer LH, Wannamethee G, Ebrahim S, Shaper AG. Serum bilirubin and risk of ischemic heart disease in middle-aged British men. Clin Chem. 1995;41: 1504–1508 7586525

[18] Kappas A. A method for interdicting the development of severe jaundice in newborns by inhibiting the production of bilirubin. Pediatrics. 2004;113: 119–123 14702459 10.1542/peds.113.1.119

[19] Ryter SW, Alam J, Choi AM. Heme oxygenase-1/carbon monoxide: from basic science to therapeutic applications. Physiol Rev. 2006;86: 583–650. 10.1152/physrev.00011.2005 16601269

[20] Barbagallo I, Nicolosi A, Calabrese G, David S, Cimino S, Madonia M, et al The role of the heme oxygenase system in the metabolic syndrome. Curr Pharm Des. 2014;20: 4970–4974 24320035 10.2174/1381612819666131206103824

[21] Jonas JC, Guiot Y, Rahier J, Henquin JC. Haeme-oxygenase 1 expression in rat pancreatic beta cells is stimulated by supraphysiological glucose concentrations and by cyclic AMP. Diabetologia. 2003;46: 1234–1244. 10.1007/s00125-003-1174-9 12898011

[22] Won KC, Moon JS, Eun MJ, Yoon JS, Chun KA, Cho IH, et al A protective role for heme oxygenase-1 in INS-1 cells and rat islets that are exposed to high glucose conditions. J Korean Med Sci. 2006;21: 418–424 10.3346/jkms.2006.21.3.418 16778382 PMC2729944

[23] Laybutt DR, Glandt M, Xu G, Ahn YB, Trivedi N, Bonner-Weir S, et al Critical reduction in beta-cell mass results in two distinct outcomes over time. Adaptation with impaired glucose tolerance or decompensated diabetes. J Biol Chem. 2003;278: 2997–3005. 10.1074/jbc.M210581200 12438314

[24] Abraham NG, Kappas A. Heme oxygenase and the cardiovascular-renal system. Free Radic Biol Med. 2005;39: 1–25. 10.1016/j.freeradbiomed.2005.03.010 15925276

[25] Chung JO, Cho DH, Chung DJ, Chung MY. The duration of diabetes is inversely associated with the physiological serum bilirubin levels in patients with type 2 diabetes. Intern Med. 2015;54: 141–146. 10.2169/internalmedicine.54.2858 25743004

[26] Onat A, Coban N, Can G, Yuksel M, Karagoz A, Yuksel H, et al Low "quotient" Lp(a) concentration mediates autoimmune activation and independently predicts cardiometabolic risk. Exp Clin Endocrinol Diabetes. 2015;123: 11–18. 10.1055/s-0034-1385922 25314652

[27] Onat A, Can G, Coban N, Donmez I, Cakir H, Ademoglu E, et al Lipoprotein(a) level and MIF gene variant predict incident metabolic syndrome and mortality. J Investig Med. 2016;64: 392–399. 10.1136/jim-2015-000003 26911630

[28] Neuzil J, Stocker R. Free and albumin-bound bilirubin are efficient co-antioxidants for alpha-tocopherol, inhibiting plasma and low density lipoprotein lipid peroxidation. J Biol Chem. 1994;269: 16712–16719 8206992

[29] Wu TW, Fung KP, Yang CC. Unconjugated bilirubin inhibits the oxidation of human low density lipoprotein better than Trolox. Life Sci. 1994;54: P477–481 8201841 10.1016/0024-3205(94)90140-6

[30] Onat A, Koroglu B, Yuksel H. The serious adjustment bias and competing outcomes in hypertriglyceridemic waist phenotype. Int J Cardiol. 2013;168: 4500 10.1016/j.ijcard.2013.06.119 23916772

