# Supplementary figures and images for: Change in Serum Bilirubin Level as a Predictor of Incident Metabolic Syndrome
Source: PLoS One. 2016 Dec 9;11(12):e0168253. doi: 10.1371/journal.pone.0168253 (PMC5148095; doi:10.1371/journal.pone.0168253)

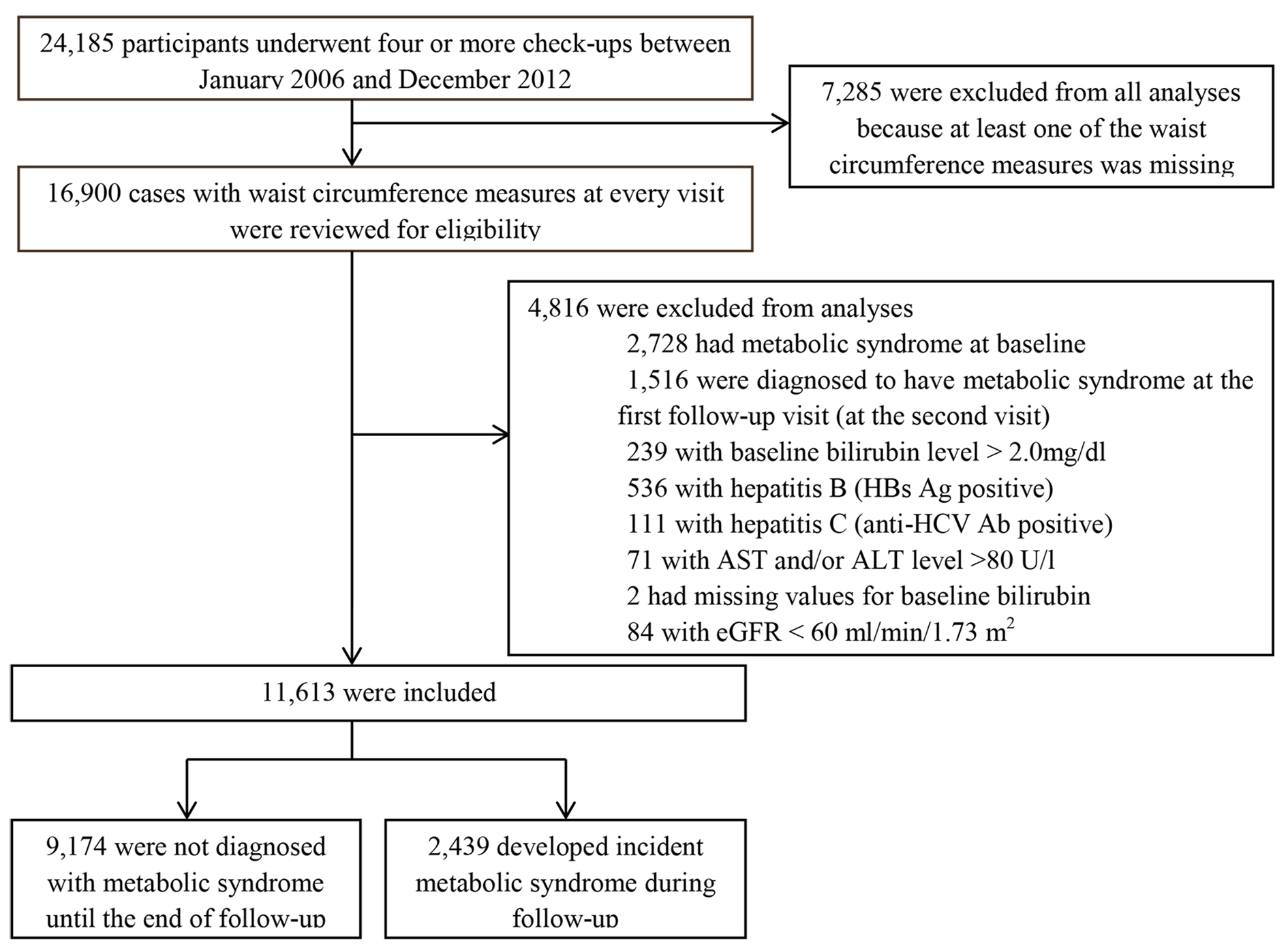

Supplement: S1 Fig — Abbreviations: HBs Ag, hepatitis B surface antigen; anti-HCV Ab, anti-hepatitis C virus antibody; AST, aspartate aminotransferase; ALT, alanine aminotransferase; eGFR, estimated glomerular filtration rate. (TIF) [file pone.0168253.s001.tif]
