# Supplementary material for: Change in Serum Bilirubin Level as a Predictor of Incident Metabolic Syndrome
Source: PLoS One. 2016 Dec 9;11(12):e0168253. doi: 10.1371/journal.pone.0168253 (PMC5148095; doi:10.1371/journal.pone.0168253)
Supplement: S1 Table — (DOCX) [file pone.0168253.s002.docx]

**S1 Table. Hazard Ratios and 95% Confidence Intervals for Incident Metabolic Syndrome according to Mean Percent Change in Bilirubin Level as a Continuous Variable in Men and Women**

|  | Men (n=6890) | | Women (n=4723) | |
| --- | --- | --- | --- | --- |
|  | Continuous variable HR (95% CI) per 1 standard deviation^a^ | *P*-value | Continuous variable HR (95% CI) per 1 standard deviation^b^ | *P*-value |
| Model 1 | 1.240 (1.192–1.290) | < 0.001 | 1.265 (1.194–1.340) | < 0.001 |
| Model 2 | 1.249 (1.201–1.299) | < 0.001 | 1.305 (1.231–1.383) | < 0.001 |
| Model 3 | 1.203 (1.156–1.251) | < 0.001 | 1.274 (1.198–1.354) | < 0.001 |
| Model 4 | 1.196 (1.144–1.249) | < 0.001 | 1.266 (1.185–1.353) | < 0.001 |
| Model 5 | 1.214 (1.152–1.279) | < 0.001 | 1.225 (1.125–1.334) | < 0.001 |

Model 1: adjusted for age, smoking status, and medication (statin and aspirin)

Model 2: adjusted for age, smoking status, medication (statin and aspirin), ALT, uric acid, eGFR, fasting glucose, and baseline diabetes mellitus prevalence

Model 3: adjusted for age, smoking status, medication (statin and aspirin), ALT, uric acid, eGFR, fasting glucose, baseline diabetes mellitus prevalence, systolic BP, waist circumference, and BMI

Model 4: adjusted for age, smoking status, medication (statin and aspirin), ALT, uric acid, eGFR, fasting glucose, baseline diabetes mellitus prevalence, systolic BP, waist circumference, BMI, and alcohol history (n = 5542 in men, n = 3936 in women)

Model 5: adjusted for age, smoking status, medication (statin and aspirin), ALT, uric acid, eGFR, baseline diabetes mellitus prevalence, systolic BP, waist circumference, BMI, and HOMA2-IR (n = 4538 in men, n = 2368 in women)

Abbreviations: HR, hazard ratio; CI, confidence interval; ALT, alanine aminotransferase; eGFR, estimated glomerular filtration rate; BP, blood pressure; BMI, body mass index; HOMA2-IR, homeostasis model assessment index 2 for insulin resistance.

^a^ 1 standard deviation in men = 32.28%

^b^ 1 standard deviation in women = 33.05%
